# High Temperature Treatment of Diamond Particles Toward Enhancement of Their Quantum Properties

**DOI:** 10.3389/fphy.2020.00205

**Published:** 2020-06-10

**Authors:** Marco D. Torelli, Nicholas A. Nunn, Zachary R. Jones, Thea Vedelaar, Sandeep Kumar Padamati, Romana Schirhagl, Robert J. Hamers, Alexander I. Shames, Evgeny O. Danilov, Alexander Zaitsev, Olga A. Shenderova

**Affiliations:** 1 Adámas Nanotechnologies, Inc., Raleigh, NC, United States; 2 Department of Chemistry, University of Wisconsin-Madison, Madison, WI, United States; 3 Department of Biomedical Engineering, University Medical Center Groningen, Groningen University, Groningen, Netherlands; 4 Department of Physics, Ben-Gurion University of the Negev, Beer-Sheva, Israel; 5 Department of Chemistry, North Carolina State University (NCSU), Raleigh, NC, United States; 6 College of Staten Island, CUNY, Staten Island, NY, United States

**Keywords:** fluorescent nanodiamond (FND), nanodiamond (ND), fluorescence, photoluminescence (PL), magnetic modulation, biosensing, photobleaching, hyperpolarization

## Abstract

Fluorescence of the negatively charged nitrogen-vacancy (NV^−^) center of diamond is sensitive to external electromagnetic fields, lattice strain, and temperature due to the unique triplet configuration of its spin states. Their use in particulate diamond allows for the possibility of localized sensing and magnetic-contrast-based differential imaging in complex environments with high fluorescent background. However, current methods of NV^−^ production in diamond particles are accompanied by the formation of a large number of parasitic defects and lattice distortions resulting in deterioration of the NV^−^ performance. Therefore, there are significant efforts to improve the quantum properties of diamond particles to advance the field. Recently it was shown that rapid thermal annealing (RTA) at temperatures much exceeding the standard temperatures used for NV^−^ production can efficiently eliminate parasitic paramagnetic impurities and, as a result, by an order of magnitude improve the degree of hyperpolarization of ^13^C via polarization transfer from optically polarized NV^−^ centers in micron-sized particles. Here, we demonstrate that RTA also improves the maximum achievable magnetic modulation of NV^−^ fluorescence in micron-sized diamond by about 4x over conventionally produced diamond particles endowed with NV^−^. This advancement can continue to bridge the pathway toward developing nano-sized diamond with improved qualities for quantum sensing and imaging.

## INTRODUCTION

The unique quantum properties of optical centers in diamond can impact technological innovation across a range of disciplines. In particular, the fluorescent, negatively charged nitrogen vacancy (NV^−^) center has been studied extensively. The electronic state of the NV^−^ center can be manipulated with external electromagnetic fields, strain fields, and temperature [1–3]. This remarkable ability to controllably manipulate and read out changes in the electronic state of the NV^−^ center make it a powerful probe for applications spanning from quantum information processing, to biology and applied medical physics. While a number of demonstrations using fluorescent diamond purely as a fluorescent label have been shown [4–6], diamond stands out against competing materials in sensing applications where the readout of fluorescence modulation is used to obtain information about the environment surrounding the optical center (e.g., biological sensing [7–11]) and can also be utilized for background-free imaging based on the magnetic modulation of the fluorescent signal [12, 13]. While bulk (electronic grade single crystalline plates) diamond containing an engineered array of subsurface NV^−^ centers has been demonstrated to advance quantum computing [14–16] and nano-NMR [17–20] applications, the use of diamond particles as nano- and microscale quantum probes holds the most promise for the future of high-tech particulate diamond applications. Fluorescent particulate diamond can enable substantial technological breakthroughs in the biological and medical disciplines, enabling analysis of local events in cells, tissue, and organisms in heterogeneous environments.

The use of fluorescent diamond particles instead of bulk single crystal materials comes with a set of challenges that must be met. Optimization of NV^−^ center quality to increase spin coherence times (T_2_) as well as spin-lattice relaxation times (T_1_) is needed, as supported by simulations [21, 22]. The inhomogeneity of color centers’ quality, content, and orientation across many particles can lead to unpredictable results across different experiments and different production batches of fluorescent diamond particles. The two main areas of focus toward improving the quality of fluorescent diamond particles involve: (1) the source of the core diamond material (synthesis) and (2) how the material is treated to optimize parameters of the color centers and particle size (processing). Control of synthesis is critical to material optimization as it can potentially allow for control of nitrogen content and distribution, metallic impurity content and distribution, and overall crystal lattice quality. While some academic effort [23–26] has been devoted toward improved synthesis, the limited access, required expertise, and high cost of growth instrumentation (specifically high-pressure high-temperature presses) has limited a greater exploration of this topic; however, this tendency is beginning to shift as the industrial synthetic diamond community has begun to take notice of the emerging potential applications of diamond particles containing color centers. Investigations into the impact of processing conditions and their influence on the quantum properties of fluorescent diamond particles have been performed and continue to be one of the key research topics [27–33]. We define processing to be any modifications made following synthesis, including irradiation, annealing, fragmentation, purification, and surface modification. Because the processing side is rich with topics for exploration, most efforts focus on this aspect.

We recently showed that high-temperature annealing can facilitate the controlled formation of a range of color centers in synthetic (HPHT) diamond particles [5, 30], allowing for a greater flexibility for the use of synthetic precursors. This is an important step since, unlike natural diamonds, synthetic diamonds afford more control over critical factors such as nitrogen content and distribution. The T_1_ relaxation times of NV^−^ centers, measured via electron paramagnetic resonance (EPR), in 20 μm particles were improved after undergoing annealing at temperature exceeding ∼1700°C [29]. These observations are consistent with what has also been observed in bulk diamond, where high-temperature annealing improved coherence times of NV^−^ centers [34, 35]. Notably, a significant dependence of the hyperpolarization capability in high-temperature annealed particles with the highest hyperpolarization enhancement for particles treated in the 1700°C−1750°C range was recently observed as well [36]. Herein, we expand our initial studies on the impact of high-temperature annealing [29, 30] and relate to the development of significantly increased diamond particle hyperpolarizability [37] to other quantum properties of NV^−^ centers in fluorescent particulate diamond, specifically magnetic modulation of the particle’s fluorescence [12, 13, 38–42]. It is envisioned that this method will facilitate identification of fluorescent diamond particle sensors in biological environments with high fluorescent background and light scattering capability. Systematically varied 20 μm samples with color centers formed by high-temperature annealing and described previously [30] were evaluated for modulation of their fluorescence under magnetic field. These samples were then characterized by EPR to learn if a correlation between fluorescence modulation and electronic spin characteristics could be observed. Notably, we observed that controlled high-temperature annealing increases the contrast achievable by magnetic modulation of fluorescence compared to the material processed by standard annealing methods. Moreover, we demonstrated that modulation occurs down to excitation with 420 nm. Finally, an initial investigation into the impact of high-temperature annealing with smaller particles sizes (∼140 nm) on another quantum property, the NV^−^ spin relaxation time, is also reported.

## EXPERIMENTAL

### Materials

Type Ib, high-pressure high-temperature (HPHT) synthesized diamond particles with sizes of ∼140 nm and 20 μm (Diamond Innovations, USA), containing ∼110 ppm of substitutional nitrogen, were used in this study. The 20 μm particles were irradiated with high-energy electrons (3 MeV) to a fluence of 1.5 × 10^19^ e/cm^2^ as previously reported [30]. These particles were then rapidly annealed at the following conditions using a previously reported [30] high-temperature annealing furnace: 1500°C/5 min., 1700°C/3 min., 1900°C/1 min., and 1740°C/8 min. Following annealing, the particles were oxidized in air at 850°C for 10–15 min. after treatment to remove graphite. The 20 μm particles were subsequently characterized by EPR and by their performance in fluorescence modulation with a magnetic field. Due to low amount of available material, the sample treated at 1740°C for 8 min. was not characterized with EPR, but was only studied for fluorescence modulation capability. The 140 nm particles were irradiated to a fluence of 1 × 10^19^ e/cm^2^ using the aforementioned high energy electrons and subsequently rapidly annealed at the following conditions (previously reported [5]): 1500°C/5min and 1700°C/3min. Graphitic carbon was subsequently removed by oxidation at 500°C for 8 h (in 2 h increments) in a Linberg Blue M Furnace followed by subsequent refluxing in a 3:1 mixture of concentrated sulfuric and nitric acids, leaving all particles with a carboxylated (-COOH) surface functional chemistry. The difference in irradiation fluence between the 20 um and 140 nm particles is related to the size-dependent survivability of particles under harsh irradiation conditions; additionally, more extensive irradiation dose were demonstrated to decrease fluorescence brightness overall [43]. The 140 nm particle series were characterized by optical relaxometry (see below). For both the 20 μm and 140 nm particle series, control samples for each were annealed using traditional annealing treatments at 850°C for 2 h under vacuum to serve as references. Oxidation of the 140 nm and 20 μm control samples to remove graphitic contributions was achieved via air oxidation at 500°C for 2 h. For the 140 nm control sample, additional treatment in concentrated sulfuric and nitric acids (3:1) was performed. A lower duration of air oxidation for the 140 nm control sample (2 h) as compared to the 8 h treatment of the RTA samples was due to the greater extent of graphitization that was observed in the RTA samples. Both samples (140 nm control and RTA 140 nm particles) exhibited a white appearance when suspended in deionized water. There was also a difference in the oxidation temperatures and times of the RTA treated 20 μm particles as compared to the control (850°C 10–15 min. and 500°C 2 h). The RTA treated 20 um particles were oxidized in the RTA furnace immediately following annealing treatment [30]. The effect produced by these differences in oxidation treatments is expected to be minimal because the color centers formed at higher temperatures during annealing are thermally stable, and the diffusion of most remaining species will be limited at these lower temperatures.

### Fluorescence Spectroscopy, Imaging, and Magnetic Modulation of Diamond Particle Fluorescence

For characterization, diamond powder was sandwiched between thin glass coverslips (Brain Research Laboratories, #4860–1). General characteristic spectra of particles were captured with an HR2000 spectrometer (Ocean Optics) with FF01–470/28 bandpass excitation and BLP01–488R longpass emission filter (Semrock) in an Olympus IX71 inverted epifluorescence microscope.

The magnetic modulation of fluorescence emission for the 20 μm series of annealed particles was then characterized under several different excitation wavelengths (420, 514, and 532 nm) under two different optical set-ups. Generally, fluorescence was collected from different spots on the sample with and without an applied magnetic field. Multiple measurements were performed for the same sample to ensure reproducibility of the results. Fluorescence spectra were recorded at the NCSU Imaging and Kinetic Spectroscopy facility (IMAKS Lab) using a custom-built Raman/fluorescence spectrometer. A Coherent Innova 70 C Spectrum ArKr laser was used to generate 514 nm excitation.

The excitation beam passed through a laser clean-up filter (MaxLine^®^ series from Semrock) and a glass plate beamsplitter at 45° incidence angle. Reflected light was focused by a 5X microscope objective with ca. 7 mm working distance onto the sample positioned on a 3-D translation stage. Fluorescence was re-collimated back by the same objective, passed through the beamsplitter and a long-pass filter (RazorEdge^®^ series from Semrock), and was focused into a 600 μm optical fiber by a 10X microscope objective. The exit end of the fiber was connected to the entrance slit of a Princeton Instruments IsoPlane SCT 320/PIXIS 100 eXelon spectrograph/CCD combo. A stack of neodymium permanent magnets (NdFeB, Grade N42 K&J Magnetics, Inc. *ca.* 300 mT) was set on a flip stage and was moved in an out at the sample backside without touching any elements of the setup or contributing any reflectance (setup shown in Figure S1). For each sample, three different locations on the coverslip were investigated, and at each position, three repetitive (*n* = 3) measurements were taken with the “magnet on” and “magnet off” states, with particles experiencing a field of ∼150 mT, which is above the saturation regime for modulation processes [44, 45]. In a second optical setup, fluorescence was measured using a custom-built inverted microscope, previously described [46]. Fluorescence excitation was provided at 532 nm (Opto Engine LLC-500mW) through a stabilizer (Thorlabs Noise Eater NEL02) or at 420 nm (PicoQuant LDH-D-C-420) and directed to the sample using a dichroic mirror (Semrock, FF-552-di02) and a 40x objective (Nikon PlanFluor, 40× 0.7 NA). Emission passed through the dichroic mirror as well as line reject filter (Semrock) or color filter to reject remaining excitation wavelengths, and was directed to a tube lens (Thorlabs) forming a real image. A set of transfer lenses either directs fluorescence to an avalanche photodiode (Excelitas SPCM-AQRH-14) through a long pass filter (Thorlabs, FELH0650), or projects the image onto the slits of a monochromator (Andor Shamrock 193i) attached to an intensified charge-coupled device (Andor iStar)Using this setup, spectra were recorded using a grating with 150 lines/mm and blazed at 300 nm. The magnetic field was produced by an electromagnet (Uxcell, 12V 50N) mounted 3 mm above the sample and powered using a source meter (Keithley, 2425 100W). In all cases, collected fluorescence spectra were subsequently analyzed to calculate the extent of fluorescence modulation with wavelength dependence modulation percentage defined as 100^∗^(I_off_-I_on_)/I_off_.

### EPR

Continuous wave X-band (9.4 GHz) EPR measurements were carried out at room temperature (RT, *T* ∼295 K) and *T* = 50 K using a Bruker EMX−220 spectrometer equipped with an Agilent 53150A frequency counter and an Oxford Instruments ESR900 variable temperature accessory. Accurate determination of *g*-factors and densities *N*_s_ of paramagnetic *S* = 1/2 species was assisted by a reference sample of a well-purified detonation nanodiamond (ND) powder with *g* = 2.0028(2) and *N*_s_ = 6.3 × 10^19^ spins/g [47]. The quantification of the NV^−^ centers content was done by comparison of the double-integrated intensities of the *g* = 4.26(1) EPR lines in all the samples studied compared with that of a fluorescent microdiamond sample FMD having NV^−^ content 5.4 × 10^17^ spin/g [48]. Electronic spin-lattice (*T*_SL_) relaxation times were evaluated by analyzing peak-to-peak amplitudes of the corresponding EPR line as a function of the incident microwave power, *P*_MW_, using the methods described elsewhere [49, 50]. EPR data processing and simulation were carried out using Bruker WIN-EPR and OriginLab software packages.

### Optical T1 Relaxometry

T_1_ relaxation times for the NV^−^ centers in the 140 nm series of particles were measured using a previously reported [51] home-built setup. The particles (850°C−2 h. control, 1500°C−5 min., 1700°C−3 min.) were prepared at ∼1 mg/mL in deionized water, and then diluted 10x with an 80:20 (v/v) mixtures of milliQ water and methanol. The methanol was added to facilitate faster solvent evaporation. After dilution, a small amount of each sample was transferred to a glass Petri dish with 4 compartments. The samples were placed in a fume hood for ∼1 h to evaporate the solvent.

For each of the three samples, a total of 10 diamond particles were characterized for T_1_ times, and a total of five repetitions (*n* = 5) were performed for each particle, thus, a total of 50 measurements were taken for each sample in the 140 nm series. In order to minimize the influence of particle aggregation, the samples were analyzed in areas of the dried particles where aggregation was qualitatively low. A small number of measurements was discarded in some cases where excessive amounts of noise, highly atypical behavior inconsistent with NV^−^ centers, or instrumentation issues (see Supplementary Information for additional discussion) were present An average T_1_ was calculated for each sample based on measurements of individual particles.

## RESULTS AND DISCUSSION

### Fluorescence Spectra of Rapid Thermal Annealing Samples

**FIGURE 1|:**
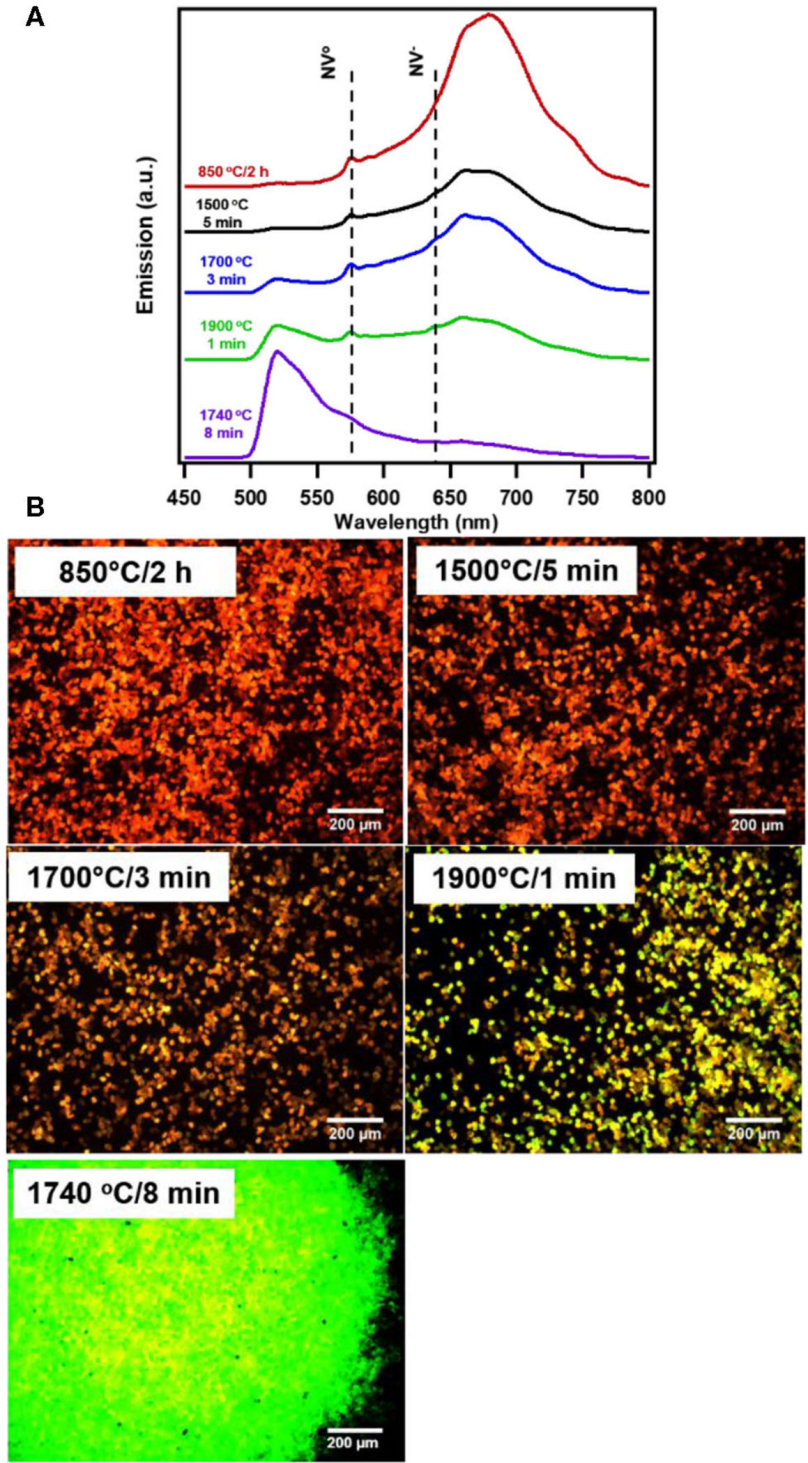
Fluorescence **(A)** spectra and **(B)** images of irradiated and typically annealed (850°C) as well as RTA treated 20 um HPHT diamond particles irradiated to 1.5 × 10^19^ e/cm^2^ fluence. The zero phonon lines for NV° and NV^−^ are indicated with a dotted line. Spectra and images were taken from bulk collection of particles under broadband illumination at 10x magnification (470/28 nm excitation and 488 nm long pass emission); images reflect true color of observed particles.

The standard annealing process (850°C/2h) of irradiated diamond particles produces particles with typical NV dominant spectra, with a broad phonon band peaking near 680 nm and low intensity below the NV^0^ zero phonon line at 575 nm (Figure 1). The key feature of rapid thermal annealing (RTA) is a fast temperature rise (at a minute-scale), which quickly achieves the temperature necessary for nitrogen diffusion (∼1500°C) while still preserving the large number of vacancies introduced by irradiation for the formation of complexes consisting of one or few nitrogen atoms and a vacancy. Samples treated at temperatures near 1500°C and above are marked with the appearance of a peak near 520 nm characteristic of H3 centers, formed by complexes of two nitrogen atoms and a vacancy (also called the NVN center). This peak, hardly seen for the sample 1500°C/5min, is more pronounced for the sample 1700°C/3min and becomes comparable in intensity with the NV peak for the sample 1900°C/1min (Figure 1A). The sample treated at 1740°C/8 min has a dominant H3 peak, while the NV peak is greatly diminished. Fluorescence micrographs of the samples study are further illustrated in Figure 1B. Notably, the 1740°C/8 min treated particles macroscopically acquired whitish color (observed in white light) as opposed to the pinkish color of particles characteristic of the other micron samples studied in this work, due to the reduced amount of NV centers.

### Magnetic Modulation of the NV Fluorescence

The magnetic sensitivity of the NV^−^ center is due to its triplet spin states (spin *S* = 1), where the brightness of fluorescence is related to the axis of quantization of the center [38]. In the absence of an external field, there are three possible orientations with respect to the symmetry axis of the NV center with two energies (m_s_ = 0 and m_s_ = ±1, separated by ∼2.87 GHz [2, 52]). Under continuous illumination, a small amount of non-radiative relaxing occurs through an intersystem crossing (ISC) from the excited triplet state to a singlet state, with greater probability of ISC for the m_s_ = ±1 state than for the m_s_ = 0 state. This difference results in less fluorescence from the m_s_ = ± 1 as compared with m_s_ = 0. ISC from the ground singlet state to the triplet favors the m_s_ = 0 state, so through repeated optical excitation and relaxation, spins accumulate in the m_s_ = 0 state resulting in spin polarization. Fluorescence modulation of NV^−^ centers arises through manipulation of theses spin populations by one of two mechanisms, either application of a microwave frequency resonant with energy gap between the spin states, depopulating the 0 spin state (so called optically detected magnetic resonance [2, 52]) or via application of a magnetic field which reorient the axis of quantization and mixes the spin states ultimately decreasing fluorescence [38, 44, 53]. Microwave-induced modulation of NV^−^ centers in bulk diamond is capable of producing fluorescence contrast up to 30% [3], however in diamond particles the degree of modulation is typically much lower. Magnetic-induced modulation provides much higher contrast and is extremely easy to implement into an imaging workflow, magnetic-modulation was assessed. Prior studies have shown that a number of factors influence the observable contrast, including particle size, magnetic field strength, and laser intensity [46]. Here these measurement factors were kept constant while the processing conditions were varied.

**FIGURE 2|:**
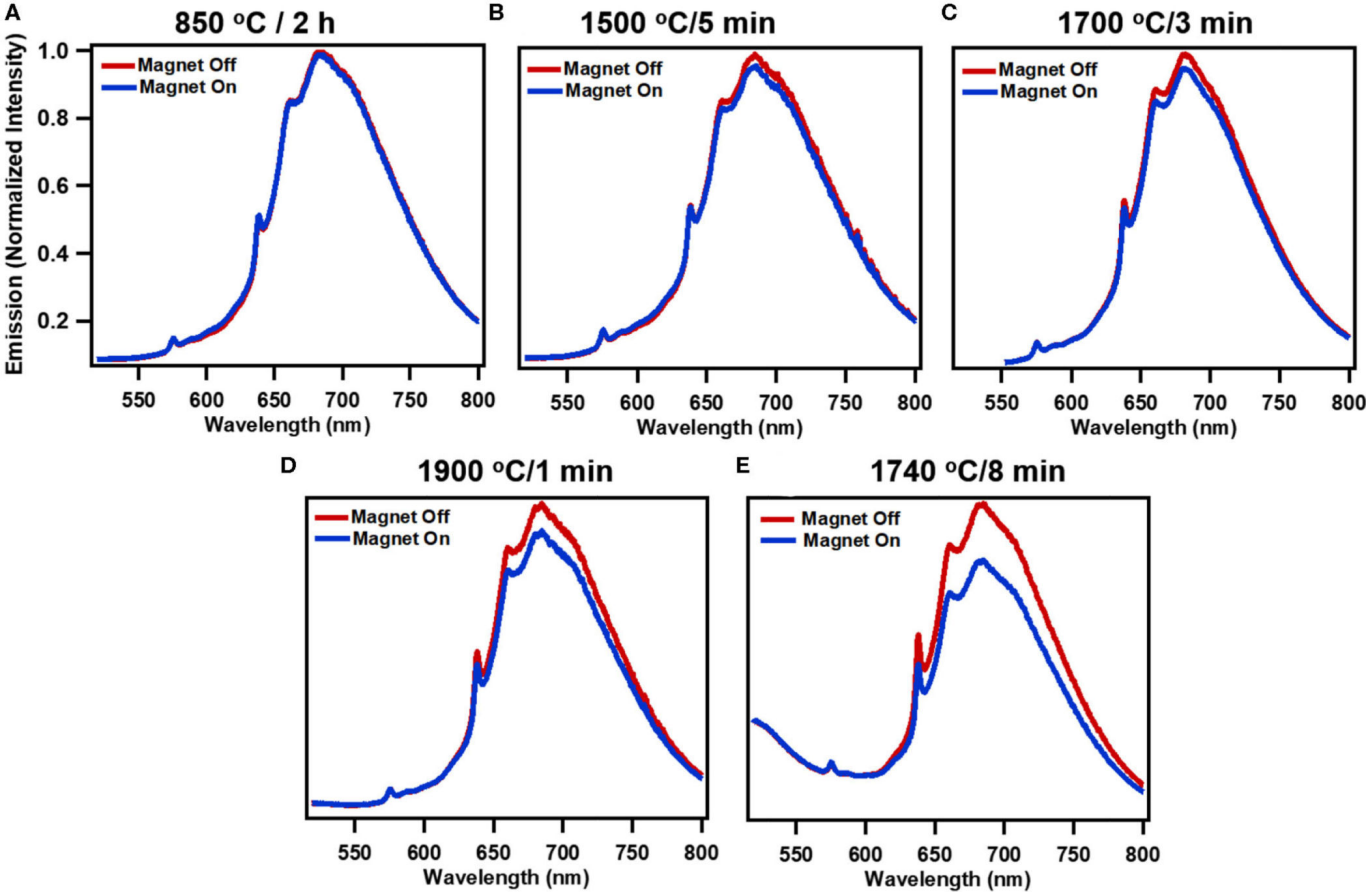
Modulation of fluorescence emission with application of static magnetic field (∼150 mT) for systematically treated samples: **(A)** standard treatment (850°C/2 h), **(B)** 1500°C/5min, **(C)** 1700°C/3min, **(D)** 1900°C/1min, and **(E)** high performing 1740°C/8min for 514 nm excitation. (Spectra are normalized averages of 3 independent measurements).

**FIGURE 3|:**
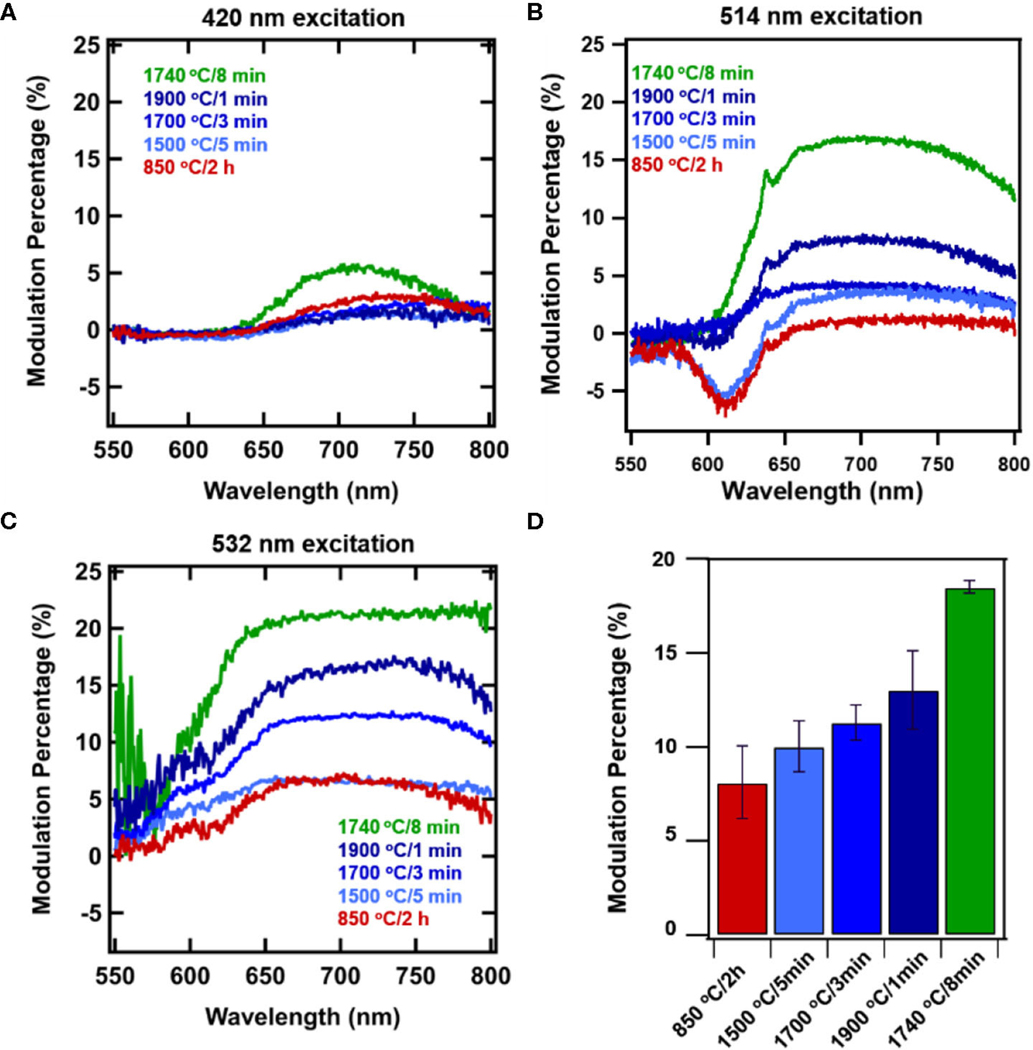
Wavelength-dependent magnetically induced fluorescence contrast due to application of a static magnetic field to systematically treated diamond samples: standard treatment (850°C/2 h), 1500°C/5min, 1700°C/3min, and 1900 C/1min, and high performing 1740°C/8min for excitation wavelengths **(A)** 420 nm, **(B)** 514 nm, and **(C)** 532 nm. Error bars represent average of 3 measurements each for 3 spots. **(D)** Modulation contrast collected for imaging under 532 excitation with 650 nm longpass.

Figure 2 shows the progressive changes in spectra due to processing conditions with and without magnetic field due at 514 nm excitation. Annealing at 850°C for 2 h represent standard conditions to anneal particles for production of NV center, and here virtually no modulation is evident peaking only at ∼2% (Figures 2A, 3B). Figures 2B–D show that as samples are RTA treated, the extent of modulation is increased with increased RTA temperature. Of the samples evaluated, treatment at 1740°C/8 min (Figure 2E) produced the greatest degree of magnetic modulation. Thus, in addition to temperature the duration of treatment plays a role (Figures 2C,E), increasing the maximum contrast to ∼16% with 514 nm excitation. This sample was identified in prior studies as having the greatest degree of ^13^C nuclear hyperpolarization arising from optical NV polarization among particles irradiated at the dosage investigated here (1.5 × 10^19^ electrons) [36]. Figures 3A–C shows modulation contrast as a function of wavelength for three excitation wavelengths. At increasing excitation wavelengths, overall modulation increases while maintaining the trends of RTA treatment. Because NV^−^is better excited at longer wavelengths, this trend is expected. The maximum increase from standard annealing is realized by treatment at 1740°C/8 min with excitation at 532 nm, increasing maximum contrast from 5 to 20%. In all excitations, modulation decreases below 650 nm due to contributions from the neutral NV^0^ center, which is not modulated by magnetic fields. Figure 3D summarizes changes in imaging contrast due to RTA for wavelengths longer than 650 nm. For use in imaging, selection of this emission window is important to both maximize contrasts, spectrometer response, and brightness [46].

As shown in Figure 1, the contribution of H3 centers increases with RTA treatment. Notably, the intensity of these H3 centers are not modulated, as is most clearly visible for 1740°C/8 min annealing (Figure 2E), where the shoulder portion below ∼550 nm is attributed to H3 centers, and no modulation is observed. Importantly, this shoulder is not observed in samples without appreciable amounts of H3 centers. Under 420 nm, there is very little modulation present in the set of RTA samples 1500–1900°C, however the sample with dominant H3 centers (1740°C) still maintains 6% modulation. This result is unprecedented, allowing for appreciable modulation in the presence of an unchanging normalization peak (H3 vs. NV^−^).

One hypothesis to explain the high modulation observed with the 1740°C annealed sample could be that the high temperature selectively anneals out lower quality NV^−^ centers. These “low quality” NV^−^ centers may be those which are located close to damaged portions of the diamond lattice (resulting from the irradiation process), those near the surface, or those near to metallic impurities or other lattice defects. Thus, the ability of an NV^−^ center to survive high-temperature annealing is possibly an indication of its resulting quality and surrounding environment. This hypothesis can be investigated in future studies with time dependent treatment at specific temperatures.

### EPR Spectra

#### Primary (S = 1/2, 3/2) Defects

**FIGURE 4|:**
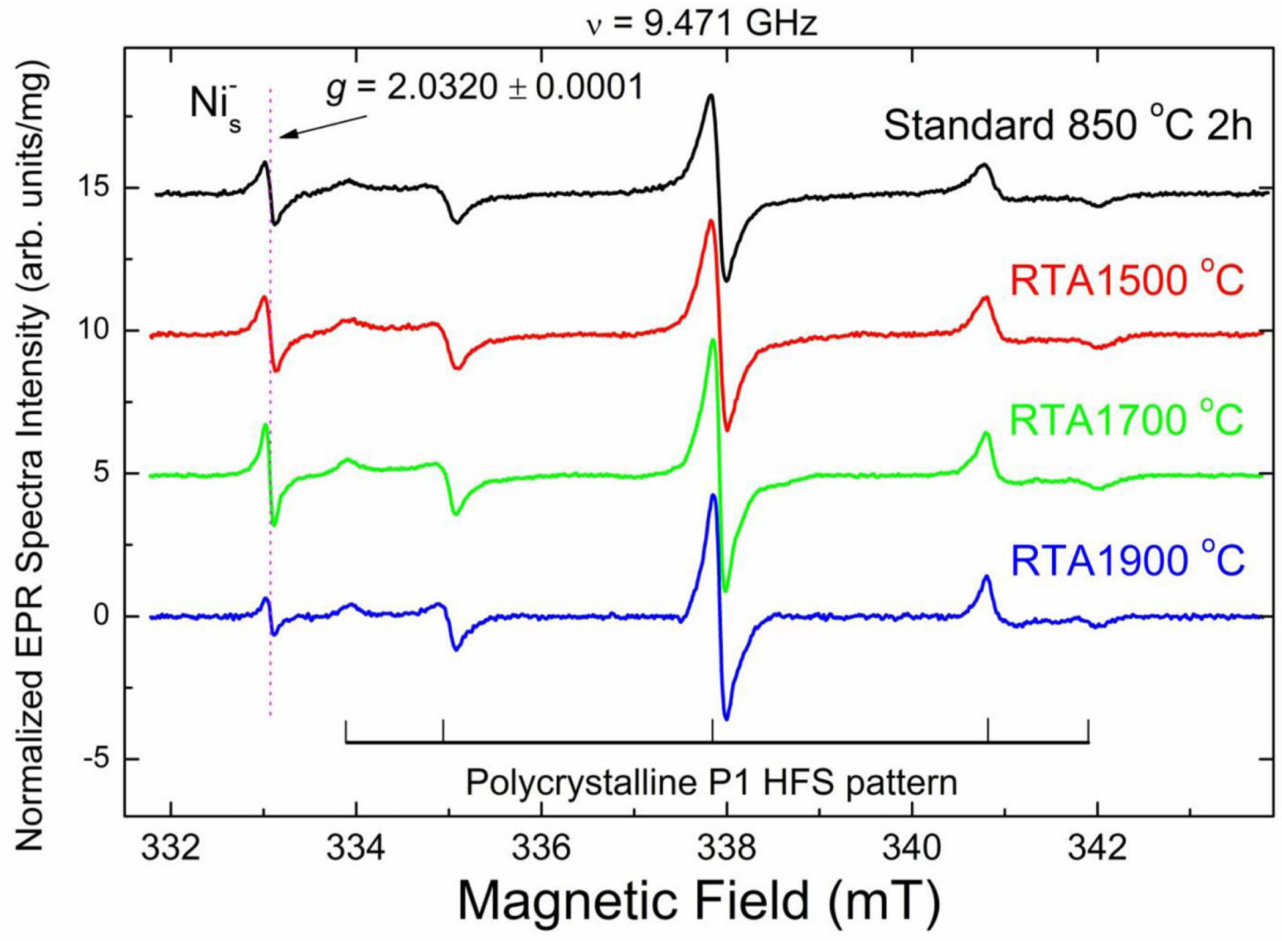
High resolution X-band RT EPR spectra of 20 μm diamond powders recorded at *T* = 50 K, *g* = 2.00 region. Spectra were recorded at the same instrumental conditions: incident microwave power *P*_MW_ = 2 μW, 100 kHz magnetic field modulation amplitude A_mod_ = 0.01 mT, receiver gain RG = 2 × 10^5^, number of scans n_acq_ = 100, microwave frequency *v* = 9.471 GHz. Intensity of each EPR signal is normalized per unit mass; spectra are vertically shifted for better presentation. Hyperfine P1 lines are partially saturated. Horizontal and vertical lines in the bottom indicate hyperfine split polycrystalline EPR pattern due to P1 centers characterized by *g*_iso_ = 2.0024 ± 0.0001, *A*_x_ = *A*_y_ = 2.96 ± 0.02 mT, *A*_z_ = 4.06 ± 0.02 mT; central hyperfine line overlaps with two singlet lines having by *g*_iso_ = 2.0028 ± 0.0001; arrow indicates position of a singlet line with *g* = 2.0320 ± 0.0001 attributed to Ni_s_^−^ defects.

Analysis of the EPR spectra of primary defects, recorded at *T* = 50 K, indicate that the sample annealed at standard conditions (850°C, 2 h) (Figure 4, black trace) contains at least 4 types of primary defects. The low- and high-field satellites in the spectra belong to the *m*_i_ = ±1 hyperfine lines of the characteristic polycrystalline pattern due to substitutional nitrogen (P1) defects in the HPHT diamond structure [54]. The central line is superposition of the sharp *m*_i_ = 0 P1-related hyperfine line with *g* = 2.0024 ± 0.0001 and two singlet signals with about the same *g*-factor *g* = 2.0028 ± 0.0001: broader and narrower, attributed to dangling bonds and vacancies (V^−^), correspondingly. The narrow line with *g* = 2.0320 ± 0.0001, observed in EPR spectra of all samples at *T* < 150 K, is attributed to negatively charged substitutional Ni center with the effective spin *S* = 3/2 (Ni_s_^−^). [55] Ni_s_^−^ content in the standard annealed sample is about 2 ppm. The starting material used contains 100 ppm of P1 centers. It was recently found that e-beam irradiation significantly reduces P1 content and increases number of V^−^ defects. Standard annealing at 850°C for 2 h drastically reduces number of V^−^ but does not affect remaining P1 centers [30]. RTA at both 1500 and 1700°C proceeds with the same tendency. Table 1 shows that the content of negatively charged vacancies decreases whereas P1 content remains the same. RTA at 1900°C practically quenches V^−^ defects and decreases the Ni_s_^−^ content, by which they become non-paramagnetic.

**FIGURE 5|:**
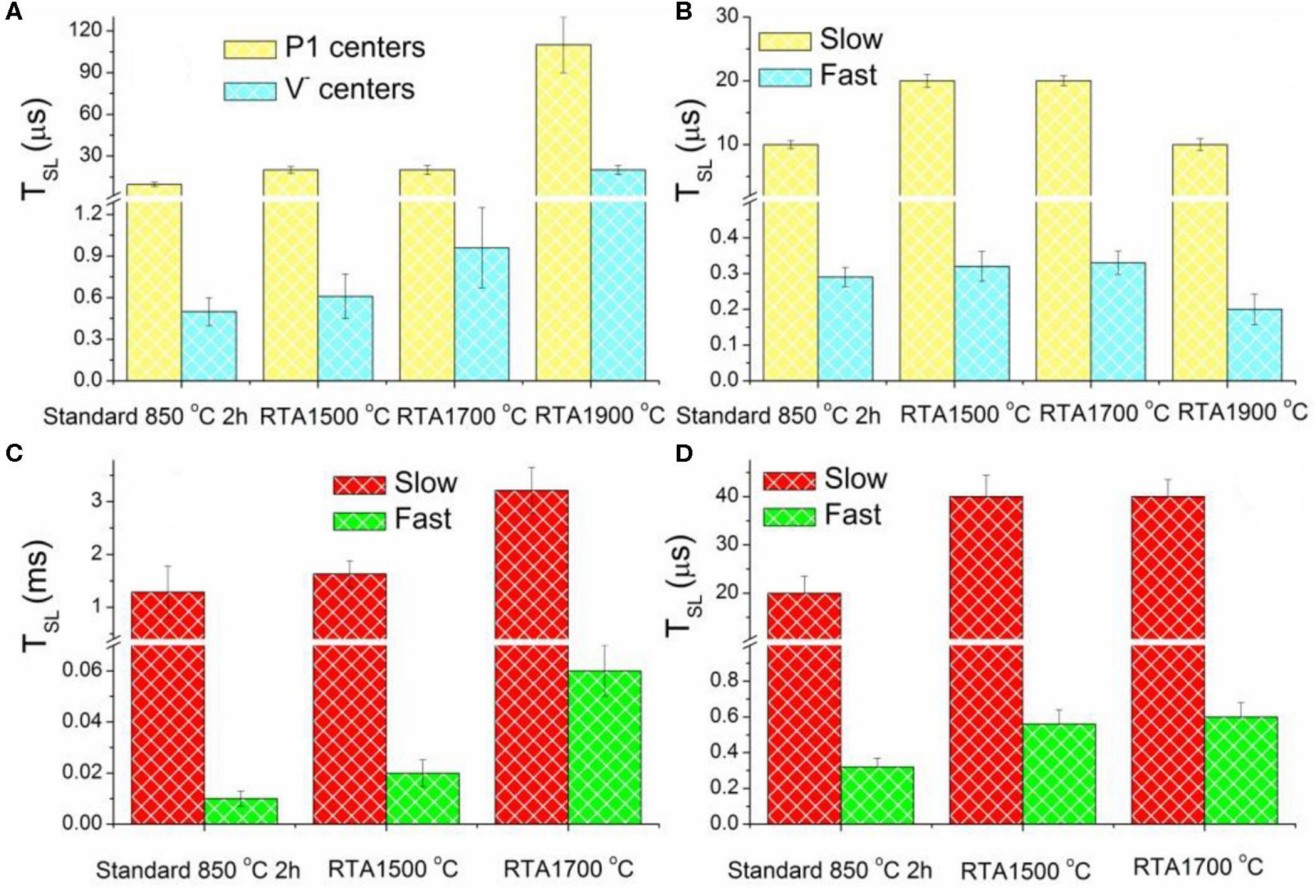
Electron spin-lattice relaxation times TSL estimated for EPR lines attributed to: **(A)** primary defects P1 and V^−^ centers (measured at RT); **(B)** Ni_s_^−^ centers (measured at *T* = 50 K); **(C)** low field “allowed” **(C)** and “forbidden” **(D)** transitions between Zeeman sublevels of the triplet NV^−^ centers (measured at RT).

Figures 5A, 2B show spin-lattice relaxation times T_SL_ estimated from the double component analysis of saturation curves recorded for primary defects for all samples of the RTA series excluding dangling bonds, which, being less abandoned, negligibly contribute to the saturation curves. RTA causes elongation of T_SL_ for both P1 and V^−^ defects—see Figure 5A, whereas T_SL_ for both slow and fast relaxing components of Ni_s_^−^ signal increase on RTA-1500°C and RTA-1700°C, then drop down on RTA-1900°C (Figure 5B). The longest T_SL_ values for P1 defects were found in the RTA-1900°C sample which may indicate, together with disappearance of V^−^ defects, some enhancement of the diamond lattice order.

#### Triplet (S = 1) Defects

Half-field EPR spectra reporting on triplet (*S* = 1) defects in the diamond samples under study have been reported in Dei Cas et al. [30]. There, lines at *g* = 4.27 ± 0.01 were reliably attributed to NV^−^ (W15) centers and additional lines with *g* < 4.27–to W16-W18 center [50]. The RTA-1500°C causes almost 30% increase of the NV^−^ content with respect to the standard annealed sample—see Table 1. The RTA-1700°C provides the NV^−^ content similar to the standard annealed sample as well as evidently decreases the content of the additional triplet centers. RTA-1900°C causes the most dramatic effect to all e-beam induced triplet defects: all triplet centers practically disappear in EPR. The intensity of the NV^−^ originated characteristic *g* = 4.270 signal drops toward the detectability level. The W16–18 signals are not detectable at all. RTA caused changes in the T_SL_ values estimated from saturation curves recorded for the low field “allowed” and half-field “forbidden” transitions in the spectra of NV^−^(W15) triplet center, demonstrating similar behavior as it was found for P1 and V^−^ centers. Thus, on increasing annealing temperature, T_SL_ for slow and fast relaxing components become significantly longer—see Figures 5C,D. The T_SL_ elongation effect reaches its maximum for RTA-1700°C but cannot be estimated for RTA-1900°C due to the NV^−^ signals intensity drops below the detection threshold. The same effect for triplet centers supports the hypothesis that RTA treatment “heals” the disorder induced in the diamond crystal lattice by intensive e-beam.

In summary, based on EPR data it may be concluded that RTA significantly reduces V^−^ content while leaving the P1 content unaffected. RTA-1500°C causes 20–30% higher NV^−^ content as compared to the sample annealed at standard conditions, while the amount of NV^−^ centers after RTA-1700°C becomes lower. Only trace amount of NV^−^ centers has been detected in the RTA-1900°C sample. RTA-1900°C annihilates triplet paramagnetic defects (W16–18) and V^−^, while reducing the amount of detected Ni^−^. At the same time RTA not only decreases the number of negatively charged vacancies, but definitely reduces the diamond lattice disorder. The latter is manifested in elongation of spin-lattice relaxation times for P1, V^−^, and NV^−^ centers. While the best performing sample for magnetic modulation of fluorescence was not investigated in the EPR, based on its loss of pinkish color, it can be assumed that NV^−^ content in this sample is at a low ppm level (see also PL spectra in Figure 1). Though a decrease in parasitic defects and improved lattice quality seem to be the most important factors in the improvement of NV^−^ quantum properties, based on the lower NV^−^ density for this sample it is possible that the reduction in NV-NV interactions also contributed to the improvement of the fluorescence contrast under magnetic modulation.

### Optical Relaxometry for Nanodiamond Particles

**FIGURE 6|:**
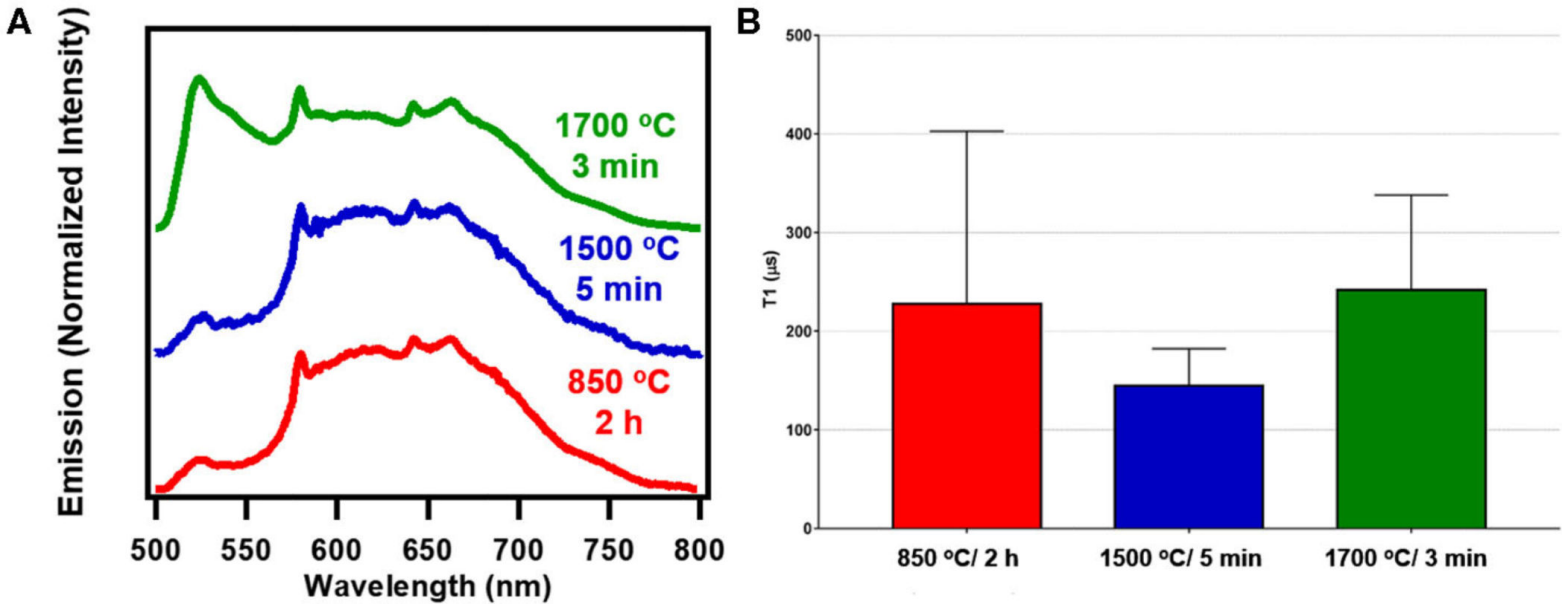
**(A)** Spectra of 140 nm fluorescent nanodiamond studied under blue broadband excitation (470/28 nm excitation and 488 nm long pass emission). **(B)** Combined average T_1_ values for 140 nm fluorescent nanodiamond particles. Statistical analysis shows that there is no significant difference between the different samples. The combined average is obtained from taking a combined curve for each particle measured and averaging the T1 values obtained from these combined curves. See Supplementary Material for additional information.

Figure 6A shows spectral profiles for nanoscale fluorescent diamond (140 nm) after RTA treatments of 1700°C/3 min or 1500°C/5 min compared to standard 850°C/2 h. Similar to bulk samples, at a greater proportion of fluorescence from H3 centers is seen at the higher treatment temperature. T_1_ optical relaxometry measurements are shown in Figure 6B along with spectral characteristics for RTA treated nanodiamond. T_1_ values for each sample in the 140 nm series of particles were determined from measurements from 10 different particles (each measured 5 times) for each sample. See Tables S1–S3 and Figures S2, S3 for all data related to these series of measurements. There appears to be a drop in the T1 values of the particles treated at 1500°C as compared to the standard annealed sample (850°C) and the 1700°C treated sample. However, a one-way ANOVA analysis performed on this data indicated that there was no statistically significant difference between samples (*P* = 0.1948). Thus, annealing has less influence on the 140 nm particles as compared to the 20 μm particles. This is possibly due to the high concentrations of spins and spin interactions between NV centers and surface species that occur at smaller particle size scales. Spin-spin interactions between multiple nearby NV centers as well as impurity spins (e.g., parasitic metallic impurities, dangling bonds in defected interface layers) may reduce T_1_ times as well. These results are perhaps not unexpected since annealing primarily impacts the bulk (lattice) of the diamond particles, and therefore has a more pronounced influence on larger particles, where “healing” of the crystalline lattice (following high energy irradiation) is more clearly observed without the influence of high concentrations of surface, impurity, or other spins in the local environment surrounding the particles. Most probably, though, higher temperature and/or increased dwell times are required, as temperatures above 1700°C were not explored in this dataset where most efficient elimination of parasitic paramagnetic defects in diamond lattice occur [36].

## CONCLUSIONS AND OUTLOOK

We determined high-temperature annealing produced a correlative effect on the achievable modulation of fluorescence by a magnetic field. The improvement of the maximum modulation contrast achievable after RTA treatment as compared to standard annealing methods rose from ∼5 to 20% under the conditions studied. The increase in modulation susceptibility of RTA treated samples increases the applicability to imaging in conditions of high fluorescent background, where fluorescence modulation of diamond can be used to perform image background subtraction [13, 41, 46]. Modulation intensity decreases with decreasing illumination wavelength below 532 nm, but is still achievable at 420 nm. Thus, the presence of dual-color emission of the non-changing H3 center provides the capability of self-calibration for imaging in conditions of quickly changing fluorescent background. EPR characterization data indicate that there is still room for further optimization of the diamond samples for quantum applications by defining the annealing conditions to preserve a larger amount of NV^−^ then what was observed for RTA-1900°C, while presumably eliminating unwanted e-beam induced paramagnetic defects, both triplets and V^−^. Currently, relaxometry data indicate that healing of the diamond lattice in 140 nm particles by annealing at maximum temperature 1700°C for 3 min explored in this study is not sufficient to provide noticeable increase in T1 relaxation time of NV- and requires further optimization.

Understanding complex intracellular mechanisms of disease progression at a cellular level is fundamental to advancing our ability to diagnose and treat illness. These results demonstrate a pathway to improve the quality of particulate fluorescent diamond particles, which have unique applications as compared to bulk diamond such as intracellular sensing [7] and as hyperpolarization agents for improving the sensitivity of nuclear magnetic resonance spectroscopy (NMR) and magnetic resonance imaging (MRI) at a cellular level [18, 56]. Owing to the high biocompatibility [1] and sensing capability, fluorescent diamond particles are ideal intracellular probes and have been demonstrated as sensors for intracellular temperature, [9, 57] radical species, [10] and pH [58]. Introducing a RTA step into the current production procedure has a high potential to further increase NV^−^ sensitivity and undoubtedly facilitate nanodiamond detection with high fidelity through selective modulation of NV^−^ fluorescence with much higher contrast over standard samples and therefore a high signal-to noise-ratio. Simultaneously, RTA treatment introduce multicolor capability to diamond particles providing an additional possibility of cross-examination for reliable detection of nanodiamonds in an environment with rapidly changing fluorescent background.

## Supplementary Material

Data Sheet

## Figures and Tables

**TABLE 1 |:** Contents of primary (*S* = 1/2, 3/2) and triplet (*S* = 1) paramagnetic centers for the samples irradiated to the fluence 1.5 × 10^19^ e/cm^2^ and annealed at different conditions.

Sample	P1, ppm^a^	V^−^,ppm^a^	Ni_s_^−^, ppm^b^	NV^−^, ppm^a^

Standard 850°C, 2 h	22	42	2.1	7.7
RTA 1500°C, 5m	26	25	3.2	9.8
RTA 1700°C, 3m	21	16	2.1	8.0
RTA 1900°C, 1m	25	0	0.9	< 0.1

a *Error does not exceed* ±*15%, data obtained at RT* [6].

b *Error does not exceed* ±*15%, data obtained at T* = *50 K.*

## Data Availability

The datasets generated for this study are available on request to the corresponding author.
