# Supplementary material for: High Temperature Treatment of Diamond Particles Toward Enhancement of Their Quantum Properties
Source: Front Phys. Author manuscript; Available in PMC 2025 Aug 13. (PMC12347466; doi:10.3389/fphy.2020.00205)
Supplement: Data Sheet [file NIHMS2043361-supplement-Data_Sheet.docx]

**Supplementary Information**

**High Temperature Treatment of Diamond Particles Toward Enhancement of Their Quantum Properties**

Marco D. Torelli,^1^ Nicholas A. Nunn,^1^ Zachary R. Jones,^2^ Thea Vedelaar,^3^ Sandeep Kumar Padamati,^3^ Romana Schirhagl,^3^ Robert J. Hamers,^2^ Alexander I. Shames,^4^ Evgeny O. Danilov,^5^ Alexander Zaitsev,^6^ Olga A. Shenderova^1^

^1^ Adámas Nanotechnologies, Inc., 8100 Brownleigh Drive, Raleigh, NC 27617, USA

^2^ Department of Chemistry, University of Wisconsin-Madison, 1101 University Avenue, Madison, WI 53706, United States

^3^ Groningen University, University Medical Center Groningen, Department of Biomedical Engineering, Antonius, Deusinglaan 1, 9713, AW, Groningen, The Netherlands

^4^ Department of Physics, Ben-Gurion University of the Negev, P.O. Box 653, 8410501 Beer-Sheva, Israel

^5^Department of Chemistry, North Carolina State University (NCSU), Raleigh, North Carolina 27695-8204, United States

^6^ College of Staten Island, CUNY, 2800 Victory Blvd., Staten Island, NY 10312, USA

*Corresponding Author E-mail: [oshenderova@adamasnano.com](mailto:oshenderova@adamasnano.com); [mtorelli@adamasnano.com](mailto:mtorelli@adamasnano.com)

*Fluorescence Modulation with Magnetic Field*

a)


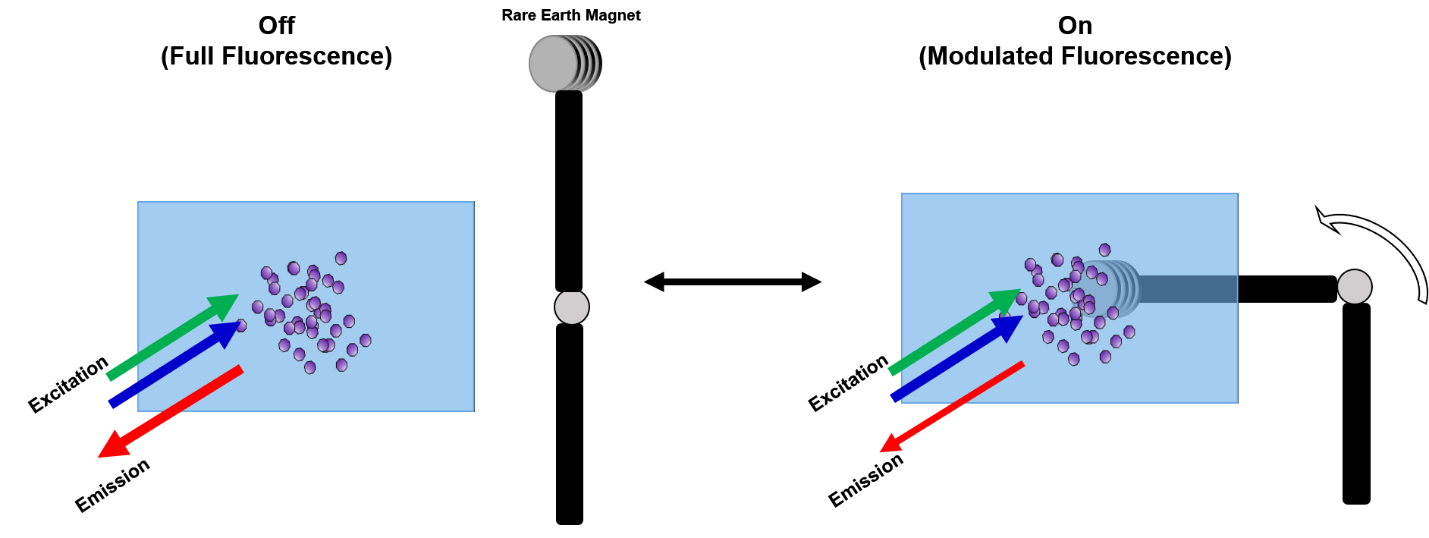


b)


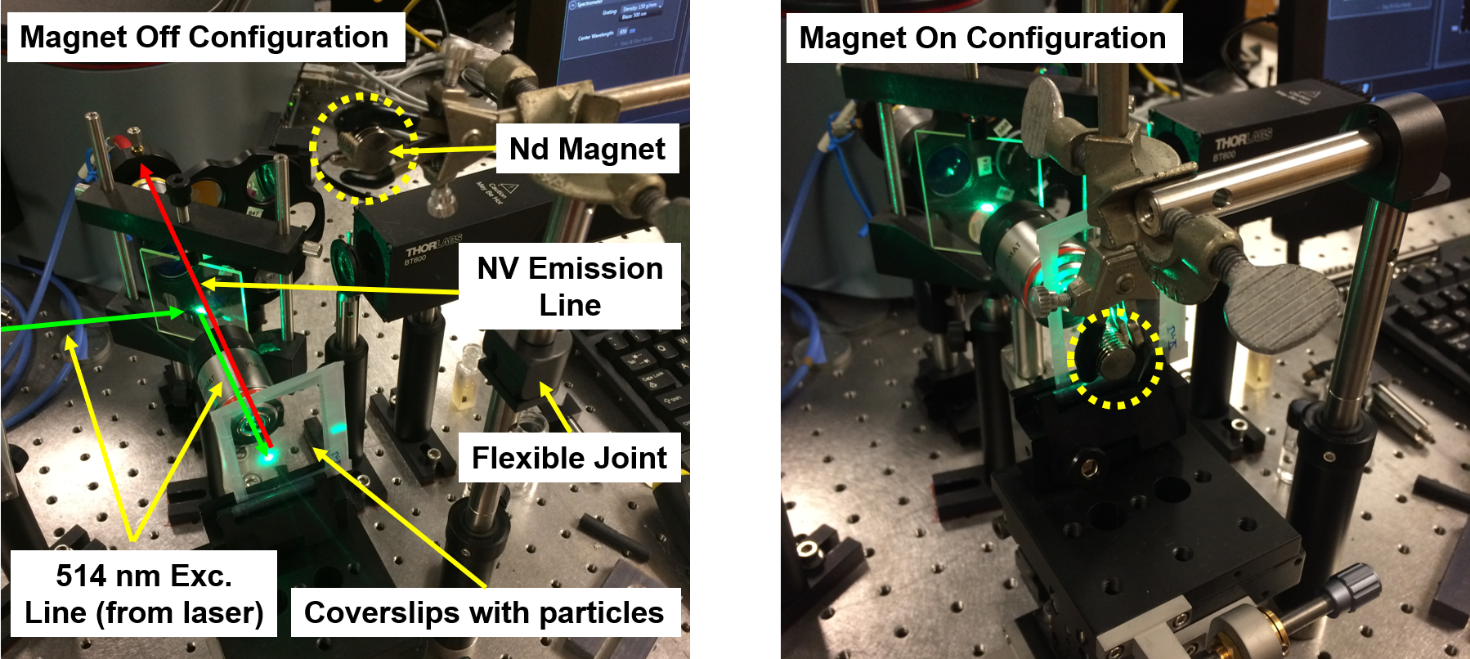


**Figure S1:** a) Cartoon schematic of setup for measurement of magnetic field induced florescence modulation in high temperature annealed diamond particles. (not to scale). B) Actual experimental setup.

*Optical Relaxometry*

The following tables (Tables S1-S3) and figures (Figures S2-S4) show the measurements collected for each of the 140 nm series annealed samples (850°C – 2 h., 1500°C – 5 min., and 1700°C – 3 min., respectively).

*850°C – 2 h. Annealing (Standard):*

Most data obtained from this sample demonstrated consistent T_1_ values (Table S1). However, there is variation across different nanodiamonds (**Figure S2**). Some measurements with T_1_ relaxation times that were on the low side (<40 µs) (increasing the amount of noise) and some that were very high (>200 µs)(increasing the error). High count rates (noise) which can impact T_1_ has several possible origins, with the most important being: (1) aggregates, (2) non-uniform particle shape, and (3) environmental noise generated from the apparatus, room, etc. Generally, the latter is less significant. Three very unusual data points are removed: 2 were removed based on showing an abnormal shape than the other curves, and one based on technical issues. The average T_1_ is obtained by averaging the values in Table S1 across all replicates for each particle. The combined curve averages the individual T_1_ curves and then fits this new curve. The value shown is the T_1_ value from this curve.

**Table S1:** T_1_ values of the particles measured from the 850°C – 2 h (standard) annealed sample

| No. of Measurement\  particle | | 1 | 2 | 3 | 4 | 5 | combined curve | average |
| --- | --- | --- | --- | --- | --- | --- | --- | --- |
| 1 | | 65.89 | 42.21 | 43.95 | 56.43 | 102.36 | 49.947 | 62.168 |
| 2 | | 305.74 | 360.76 | 582.96 | 342.65 | 231.82 | 317.36 | 364.786 |
| 3 | | 37.09 |  | 89.9 |  | 45.45 | 47.58 | 57.48 |
| 4 | | 223 | 117.56 |  | 423.26 | 222.64 | 198.45 | 246.615 |
| 5 | | 23.01 | 26.04 | 23.48 | 23.89 | 23.24 | 24.187 | 23.932 |
| 6 | | 470.01 | 593.63 | 356.61 | 424.61 | 467.25 | 458.53 | 462.422 |
| 7 | | 347.09 | 316.68 | 293.91 | 232.11 | 238.33 | 300.18 | 285.624 |
| 8 | | 124.35 | 205.27 | 149.87 | 106.24 | 99.33 | 128.64 | 137.012 |
| 9 | | 261.77 | 356.44 | 248.42 | 279.02 | 161.7 | 217.84 | 261.47 |
| 10 | | 542.36 | 698.71 | 493.59 | 437.29 | 465.6 | 532.63 | 527.51 |
|  |  |  |  |  |  |  |  |  |


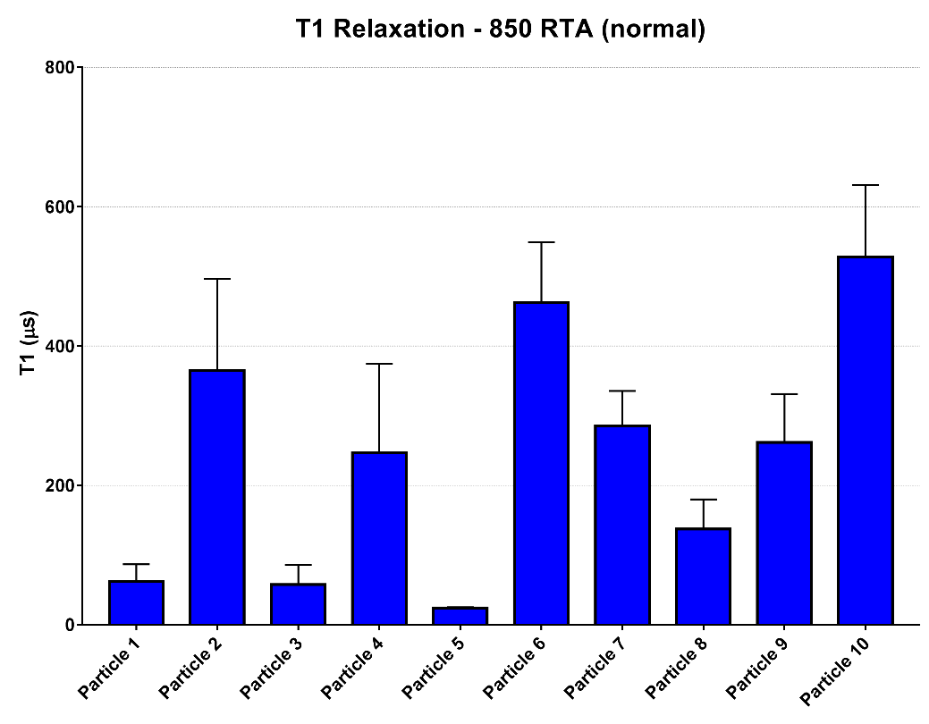


**Figure S2:** Average T_1_ values of different nanodiamonds of the 850°C – 2 h annealed sample.

*1500°C – 5 min. Rapid Thermal Annealing (RTA):*

The average T_1_ for these particles was lower than for the 850°C – 2 hr. annealed sample. Based on this, even on low counts (<40), a clear build up in the pulses could be observed due to the large difference between the pulses. A few samples had to be removed from the analysis based on either bad fitting or too little counts. All the T_1_ values for each measurement are shown in **Table S2**. The average T_1_ based on averaging the individual T_1_ values is shown in **Figure S3**. Both the table and figure show significant modulation in the T_1_ values calculated for the same particle. The average T_1_ and combined curve averages are calculated in the same way as the 850°C and 1700°C samples.

**Table S2:** T_1_ values of the particles measured from the 1500°C – 5 min. RTA sample

| No. of Measurement\  particle | | 1 | 2 | 3 | 4 | 5 | combined curve | average |
| --- | --- | --- | --- | --- | --- | --- | --- | --- |
| 1 | |  | 74.46 | 93.78 | 109.14 | 131.1 | 98.179 | 102.12 |
| 2 | | 207.71 | 159.92 | 263.6 | 247.05 | 226.78 | 221.68 | 221.012 |
| 3 | | 115.1 | 133.49 | 154.63 | 238.83 | 156.43 | 145.74 | 159.696 |
| 4 | | 100.25 | 177 | 129.44 |  | 178.37 | 149.34 | 146.265 |
| 5 | | 180.58 | 404.16 |  | 132.88 | 161.31 | 150.01 | 219.7325 |
| 6 | |  | 199.05 | 85.45 | 151.8 | 406.48 | 182.48 | 210.695 |
| 7 | | 197.09 | 97.32 | 94.51 | 159.77 | 133.49 | 98.619 | 136.436 |
| 8 | | 133.05 | 86.94 | 143.23 | 78.01 | 142.2 | 114.45 | 116.686 |
| 9 | | 90.59 | 182 | 221.52 | 93.79 | 142.95 | 138.84 | 146.17 |
| 10 | | 180.64 | 120.88 | 203.84 | 150.42 | 127.36 | 147.89 | 156.628 |
|  |  | | | | | | | |


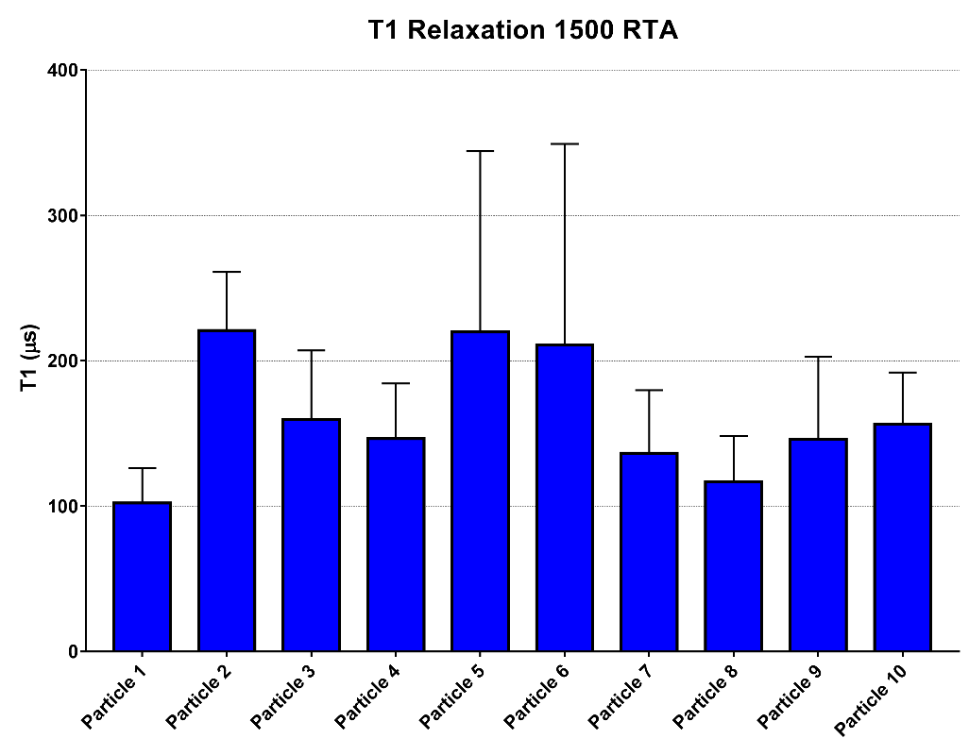


**Figure S3**: Average T_1_ values of different nanodiamonds of the 1500°C – 5 min. RTA sample.

*1700°C – 3 min. Rapid Thermal Annealing:*

Data obtained from this sample was lower in quality compared to 850°C and 1500°C samples. A few measurements (**Figure S4**) as well as a few nanodiamonds measurements (**Table S3**) were omitted because of too little counts and irregular pulse shapes, as they hinder proper analysis. Three particles were removed completely, and four data counts on different particles are excluded. The T_1_ values for each measurement of the remaining particles are shown in **Table S3** and the average T_1_ values based on averaging the individual T_1_ values is shown in **Figure S4**. The average T_1_ value is obtained by averaging the T_1_ values in the table. The average T_1_ and combined curve averages are calculated in the same way as the 850°C and 1500°C samples.

**Table S3:** T_1_ values of the particles measured from the 1700°C – 3 min. RTA sample

| No.. of Measurement\  particle | | 1 | 2 | 3 | 4 | 5 | combined curve | average |
| --- | --- | --- | --- | --- | --- | --- | --- | --- |
| 1 | | 556.74 | 115.51 | 238.28 | 478.33 | 149.67 | 215.34 | 307.706 |
| 2 | |  |  | 521.49 | 197.6 | 365.93 | 357.17 | 361.6733 |
| 3 | | 278.21 |  | 228.53 | 125.97 | 234.75 | 178.59 | 216.865 |
| 4 | | 557.35 | 180.53 |  | 731.29 | 269.03 | 354.22 | 434.55 |
| 5 | | 102.69 | 575.08 | 394.05 | 341.07 | 476.33 | 303.63 | 377.844 |
| 9 | | 228.19 | 181.53 | 191.57 | 119.08 | 152.58 | 164.07 | 174.59 |
| 10 | | 110.84 | 95.35 | 115.68 | 137.61 | 96.48 | 118.39 | 111.192 |
|  |  | | | | | | | |


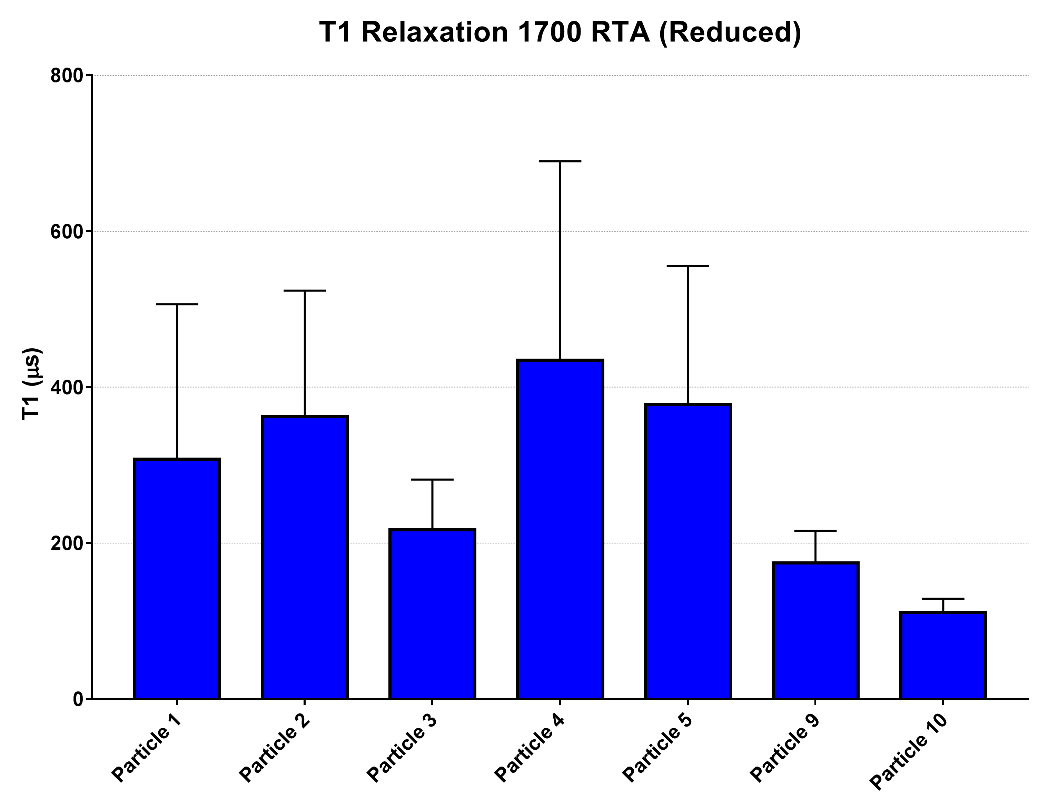


**Figure S4**: AverageT_1_ values of different nanodiamonds of the 1700°C – 3 min. RTA sample.
